# Long-Standing Unilateral Verrucous Papules of the Hand Revealing Hypertrophic Lichen Planus: A Diagnostic Challenge

**DOI:** 10.7759/cureus.105043

**Published:** 2026-03-11

**Authors:** Younes Tamim, Yassine Berrada, Mariame Meziane, Benzekri Laila, Karima Senouci

**Affiliations:** 1 Dermatology, Ibn Sina University Hospital Center, Mohammed V University, Rabat, MAR

**Keywords:** clinicopathologic, correlation, hand, hypertrophic, lichen, planus, pseudoepitheliomatous, unilateral, verrucous, wrist

## Abstract

Lichen planus (LP) is an inflammatory disorder that can involve the skin, nails, hair follicles, and mucous membranes and may present with diverse clinical morphologies that mimic other dermatologic conditions. Hypertrophic (verrucous) LP is a chronic variant characterized by hyperkeratotic lesions that most often affect the lower extremities, which makes isolated hand involvement diagnostically challenging. We report a 65-year-old man with skin-colored, dermally embedded verrucous papules confined to the right hand with extension to the wrist, evolving for more than 40 years. He denied pruritus, pain, and functional limitation. Mild nail atrophy was noted, and there was no mucosal involvement. Skin biopsy revealed pseudoepitheliomatous acanthosis consistent with cicatricial-stage verrucous LP. Treatment with a very potent topical corticosteroid (clobetasol propionate) under occlusion was initiated, though adherence was poor, and weekly trichloroacetic acid sessions were planned. Clinicopathologic correlation supported the diagnosis in this atypical unilateral presentation and guided management and follow-up.

## Introduction

Lichen planus (LP) is a chronic inflammatory disorder that may involve the skin, oral and genital mucosa, scalp, and nails [1,2]. Classic cutaneous LP is commonly described by the “six Ps” (planar/flat-topped, purple/violaceous, polygonal, pruritic, papules, and plaques) [1,2]. Hypertrophic (verrucous) LP is a chronic variant characterized by hyperkeratotic papules or plaques that most often affect the lower extremities [1,2]. When LP presents at atypical sites, including the hands, it may mimic other verrucous or hyperkeratotic conditions such as viral warts, lichen simplex chronicus, or keratinizing neoplasms, creating diagnostic uncertainty [3,4]. In addition, pseudoepitheliomatous hyperplasia in hypertrophic LP can histologically resemble well-differentiated squamous cell carcinoma, making clinicopathologic correlation essential [4-6]. We report an unusual case of long-standing, strictly unilateral verrucous papules confined to the right hand with extension to the wrist, highlighting this uncommon presentation and its diagnostic pitfalls.

## Case presentation

A 65-year-old man with no significant past medical history presented with a long-standing eruption on the right hand, evolving for more than 40 years. The lesions initially appeared as small, skin-colored papules and gradually increased in number over time. He denied pruritus, pain, bleeding, or functional impairment, and no other symptoms were reported.

**Figure 1:**
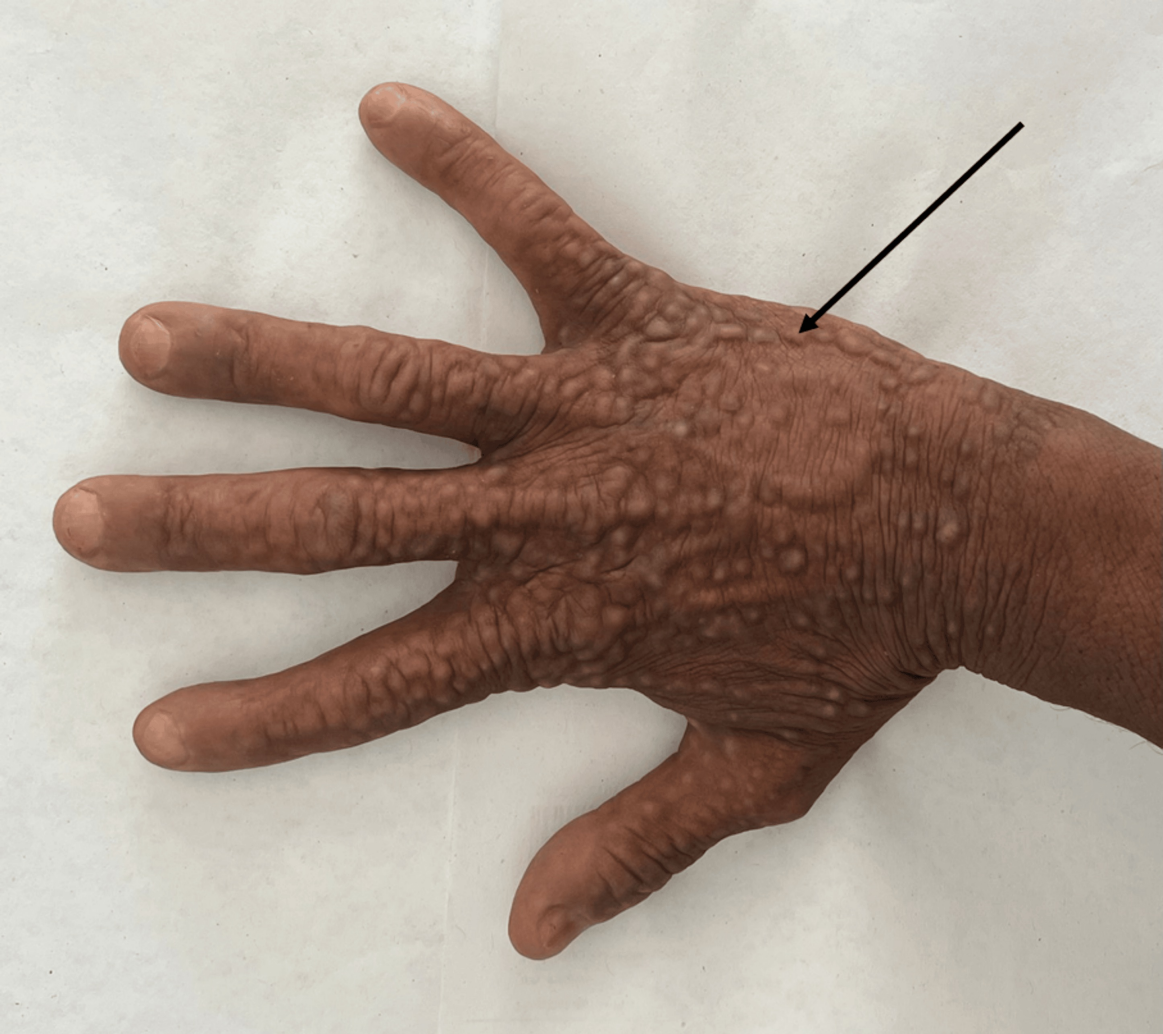
Dorsal view of the right hand Multiple skin-colored, firm, verrucous papules confined to the dorsum of the right hand.

Cutaneous examination revealed multiple skin-colored to whitish, firm, verrucous papules with a dermally embedded appearance, coalescing in some areas. The eruption was strictly unilateral (Figure 1).

**Figure 2:**
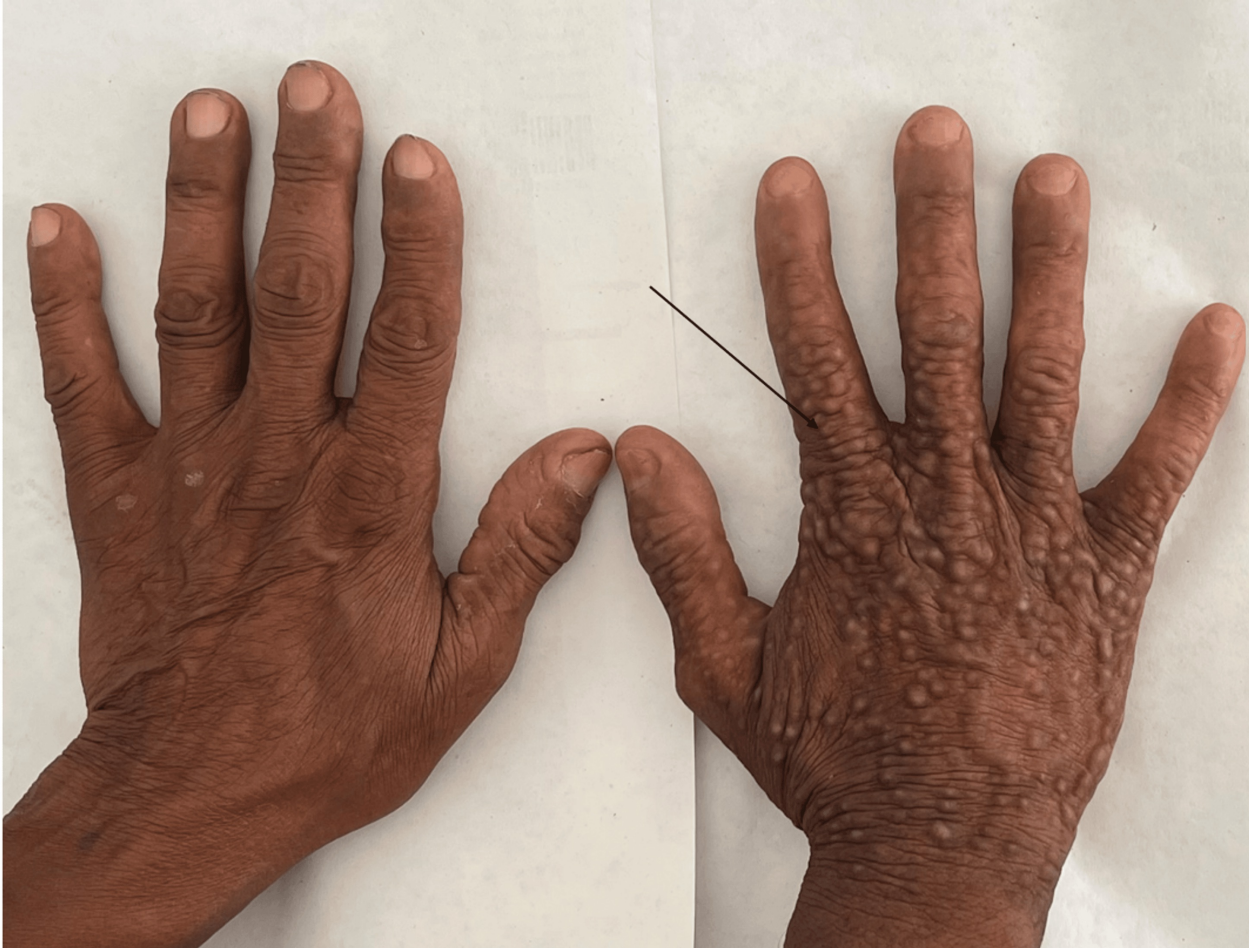
Dorsal view of both hands showing strictly unilateral involvement Multiple skin-colored, firm, verrucous papules confined to the dorsum of the right hand, with no comparable lesions on the left.

A dorsal view of both hands showed involvement confined to the right hand, with no comparable lesions on the left (Figure 2).

**Figure 3:**
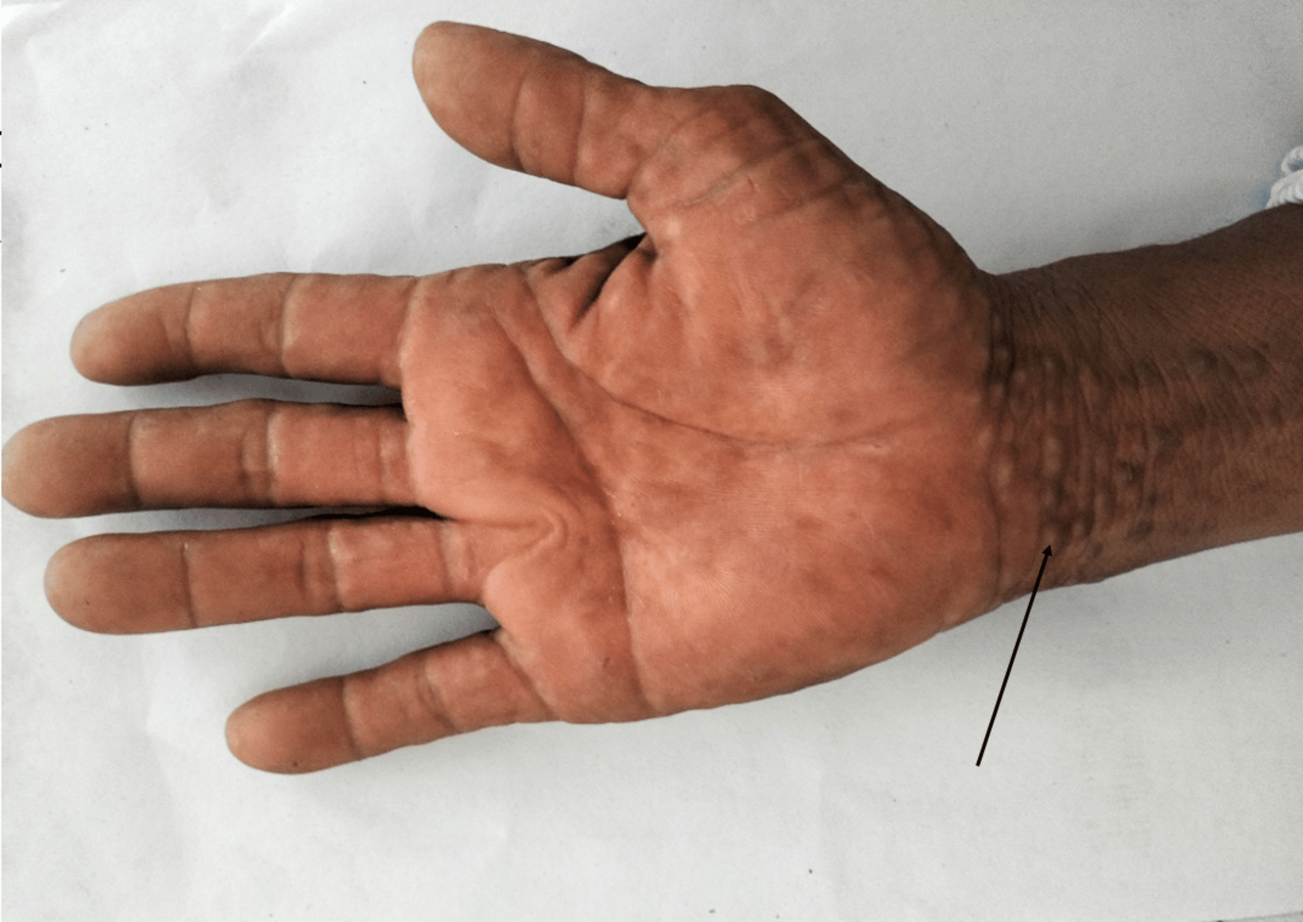
Palmar view of the right hand Skin-colored verrucous papules with a dermally embedded appearance extending to the wrist.

Palmar view of the right hand demonstrating extension of the lesions to the wrist (Figure 3).

Nail examination showed mild atrophy of the nail plate on the affected side. No oral, genital, or other mucosal lesions were identified. No lymphadenopathy was noted.

**Figure 4:**
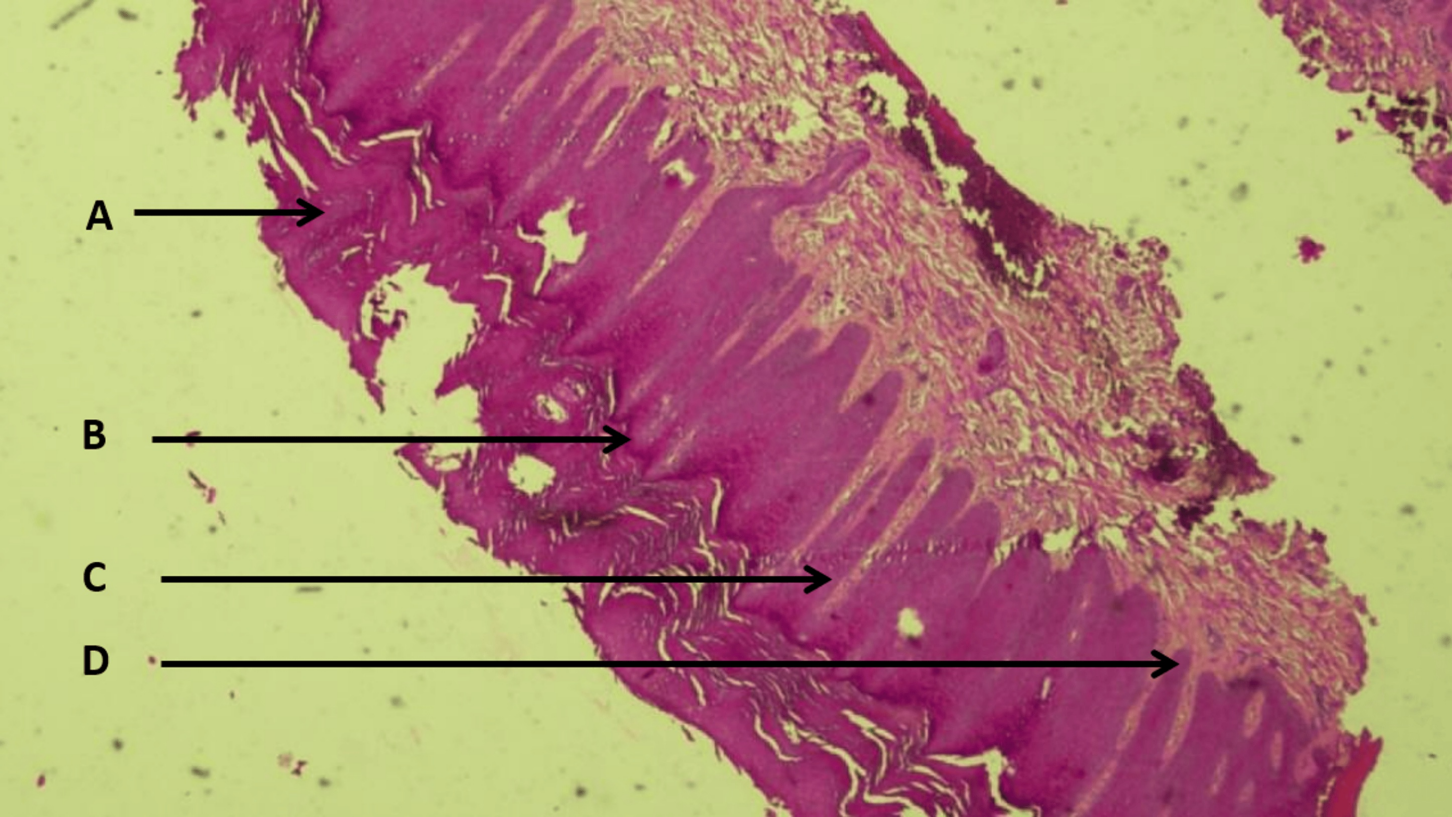
Histopathology showing features consistent with verrucous (hypertrophic) LP in the cicatricial stage (H&E, ×40) (A) Orthokeratotic hyperkeratosis. (B) Prominent hypergranulosis with papillomatous surface change. (C) Regular acanthosis with pseudoepitheliomatous pattern. (D) Intact basement membrane. H&E: hematoxylin and eosin stain; LP: lichen planus

A skin biopsy was performed. Histopathology showed pseudoepitheliomatous acanthosis consistent with verrucous LP in the cicatricial stage. The term “cicatricial stage” was used in the pathology report; however, a detailed description of dermal findings (e.g., the extent of lichenoid infiltrate or fibrosis) was not available. Additional features included orthokeratotic hyperkeratosis, prominent hypergranulosis with papillomatous surface change, regular acanthosis, and an intact basement membrane (hematoxylin and eosin (H&E), ×40) (Figure 4).

Treatment was initiated with a very potent topical corticosteroid under occlusion (clobetasol propionate; Dermoval® cream). Instructions regarding treatment use and occlusion were explained to the patient; however, adherence was suboptimal because the prescribed regimen was not followed consistently. Weekly trichloroacetic acid (TCA) sessions were proposed as adjunctive therapy, but the patient declined them because the lesions were asymptomatic. After three weeks of follow-up, the lesions remained stable, with no clinical improvement.

## Discussion

Hypertrophic (verrucous) LP (HLP) is a chronic variant of LP characterized by thickened, hyperkeratotic papules or plaques, most commonly involving the lower extremities and often associated with pruritus [1,2]. The present case is diagnostically challenging because the eruption was strictly unilateral, confined to the right hand with extension to the wrist, and had an exceptionally long duration (>40 years) with minimal symptoms. Such an atypical distribution can delay recognition and broaden the differential diagnosis, particularly on the hand where several verrucous disorders are far more common [3,4].

Clinically, long-standing verrucous papules of the hand may resemble viral warts, lichen simplex chronicus, porokeratosis, and other hyperkeratotic dermatoses [1,4]. Prurigo nodularis can also enter the differential diagnosis, but it is typically intensely pruritic, unlike in our patient [1,2]. In an indolent unilateral eruption persisting for decades, biopsy is therefore essential to avoid prolonged misclassification and inappropriate treatment.

A well-recognized issue in HLP is the presence of pseudoepitheliomatous hyperplasia (pseudoepitheliomatous acanthosis), which may create diagnostic uncertainty if interpreted without adequate clinicopathologic correlation [4-6]. In our case, histology supported verrucous LP in the cicatricial stage, and the available microscopic features, orthokeratotic hyperkeratosis, prominent hypergranulosis with papillomatous surface change, regular acanthosis, and an intact basement membrane, were consistent with a chronic inflammatory verrucous LP process. Importantly, the very slow evolution, lack of pain or ulceration, and overall stability favored a benign inflammatory disorder rather than a neoplastic process, illustrating the value of correlating clinical behavior with histopathology in verrucous presentations [4-6].

For localized cutaneous LP, high- to very-high-potency topical corticosteroids are first-line therapy, and hypertrophic lesions may benefit from occlusion to enhance penetration [1,2]. Intralesional corticosteroids can be considered for thick or treatment-resistant lesions [1]. In this patient, clobetasol under occlusion was initiated; however, poor adherence likely limited effectiveness, highlighting a practical barrier frequently encountered in chronic dermatoses. For markedly hyperkeratotic or verrucous lesions, adjunctive approaches have been reported, including systemic retinoids in selected refractory cases [7]. The planned weekly TCA sessions represent a local adjunctive strategy, with follow-up planned to assess efficacy and tolerability.

Although HLP is generally benign, long-standing verrucous lesions warrant periodic clinical follow-up, primarily to monitor response and to reassess if morphology changes. Repeat biopsy is reserved for lesions that develop new warning signs such as rapid enlargement, persistent ulceration, bleeding, or pain [8,9]. This case highlights that HLP can present as a bizarre, long-standing unilateral hand-and-wrist eruption with minimal symptoms; recognizing this possibility and relying on clinicopathologic correlation can prevent misdiagnosis as more common verrucous hand conditions and guide appropriate conservative management [4-6,10].

## Conclusions

HLP is typically a chronic hyperkeratotic variant that most often affects the lower extremities. This case illustrates a rare and diagnostically challenging presentation with strictly unilateral verrucous papules confined to the hand and wrist, evolving for more than four decades with minimal symptoms. In such atypical verrucous eruptions of the hand, skin biopsy and careful clinicopathologic correlation are essential to establish the diagnosis and avoid misclassification. Very potent topical corticosteroids under occlusion remain a cornerstone of treatment for localized disease, with adjunctive therapies considered based on lesion thickness and treatment response, along with periodic follow-up to monitor evolution and therapeutic outcomes.
